# Novel Diagnostic Value of Driver Gene Transcription Signatures to Characterise Clear Cell Renal Cell Carcinoma, ccRCC

**DOI:** 10.3389/pore.2022.1610345

**Published:** 2022-05-02

**Authors:** Zsuzsanna Ujfaludi, Levente Kuthi, Gabriella Pankotai-Bodó, Sarolta Bankó, Farkas Sükösd, Tibor Pankotai

**Affiliations:** Albert Szent-Györgyi Clinical Center, Albert Szent-Györgyi Medical School, Institute of Pathology, University of Szeged, Szeged, Hungary

**Keywords:** ccRCC, gene expression signature, tumour profiling, driver gene, statistical analyses

## Abstract

Routine molecular tumour diagnostics are augmented by DNA-based qualitative and quantitative molecular techniques detecting mutations of DNA. However, in the past decade, it has been unravelled that the phenotype of cancer, as it’s an extremely complex disease, cannot be fully described and explained by single or multiple genetic variants affecting only the coding regions of the genes. Moreover, studying the manifestation of these somatic mutations and the altered transcription programming—driven by genomic rearrangements, dysregulation of DNA methylation and epigenetic landscape—standing behind the tumorigenesis and detecting these changes could provide a more detailed characterisation of the tumour phenotype. Consequently, novel comparative cancer diagnostic pipelines, including DNA- and RNA-based approaches, are needed for a global assessment of cancer patients. Here we report, that by monitoring the expression patterns of key tumour driver genes by qPCR, the normal and the tumorous samples can be separated into distinct categories. Furthermore, we also prove that by examining the transcription signatures of frequently affected genes at *3p25*, *3p21* and *9p21.3* genomic regions, the ccRCC (clear cell renal cell carcinoma) and non-tumorous kidney tissues can be distinguished based on the mRNA level of the selected genes. Our results open new diagnostics possibilities where the mRNA signatures of tumour drivers can supplement the DNA-based approaches providing a more precise diagnostics opportunity leading to determine more precise therapeutic protocols.

## Introduction

Kidney and renal pelvis cancers were the 16th most frequent cancer types globally, taking 2.2% of all newly diagnosed cases and near 180,000 deaths in 2020 [1]. These disorders predominantly occur in older adults, typically between ages 50 and 70, and 1 out of 48 men and 1 in 83 women develop them during their lifetime. Renal cell carcinomas (RCCs) represent almost 90% of kidney and renal pelvis cancers possessing many subtypes distinguished by histological features [2]. Clear-cell renal cell carcinoma (ccRCC) is the most common among the others, accounting for approximately 70%–80% of the RCCs [3].

The first identified oncogenic driver of ccRCC was the tumour suppressor *von-Hippel-Lindau* (*VHL*) gene [4], followed by the further revelation of *SET domain containing 2, histone lysine methyltransferase (SETD2)*, *BRCA1 associated protein 1 (BAP1)* and *polybromo 1 (PBRM1)* among the others. *VHL* is the most frequently inactivated of these, followed in incidence by *PBRM1* (40%–50%), *SETD2* (12%) and *BAP1* (10%) [5–7]. Interestingly, all of these genes are located on the short arm of the 3rd chromosome, *VHL* is encoded in locus 25, the three other genes are in locus 21. Multiregional next-generation DNA-sequencing cohort studies, like that of TRACERx consortia, revealed the astounding complexity of ccRCC. Tracing the evolution, up to 30 driver events were observed in each tumour with subsequent clonality. Trujalic and colleagues also showed that this clonal evolution impacts the prognosis of the disease as having more than 10 subclonal driver events besides broad parallel progression was correlated with rapid tumour growth and metastases development. Copy number and point mutations of early tumorigenic events are fixed, and mapping these, the authors identified seven evolutionary subtypes of ccRCC [8]. The other main conclusion of the cohort was that chromosome complexity strongly correlated with the tendency of metastases, and the genetic alteration of chromosome *9p* was a driver for metastasis and ccRCC related mortality [9]. In addition, the promoter methylation of transcript variants of *CDKN2A*, including *p14* ^*ARF*^, encoded on *9p21.3*, was recently shown to be correlated with RCC tumorigenesis [10].

From the middle 90s, Sanger sequencing, PCR-based mutation and microsatellite testing and comparative genomic hybridisation techniques were commonly used in clinical genetic investigation. However, from 2012 the use of next-generation sequencing (NGS) approaches gradually gained round over these. With the rapid development of the NGS technologies, working and analyses pipelines were getting more cost-effective, reliable, and fast and further, making an essential contribution to identifying so-called risk genes in oncology. Based on the emerging knowledge of the molecular background of different tumour types, gene panels were developed for clinical testing [11, 12]. Upon these panels, by targeted re-sequencing of selected high-, moderate- and low-risk genes, single nucleotide polymorphisms (SNVs), indels, among the others, can be evaluated on DNA derived from patient surgical samples, biopsies and even from cell-free DNA of blood sera.

However, a quite high proportion of the patients reveal mutation-negative or undiagnosed [13, 14], besides the identification of germline or somatic mutations does not provide any information about the biological manifestation of those. RNA testing of tumour patient samples is also promising; nonetheless, the NGS-based approaches mainly focus on reconning splice variants, the abundance of pathogenic RNA, and non-coding RNAs [15]. Also, many studies were conducted to develop panels for RNA testing in oncology, mainly by identifying differentially expressed genes using microarray and RNA-seq techniques. Efforts to seek cheap and quick laboratory approaches that provide reliable tumour diagnoses are essential to support the realisation of personalised medicine. Here we show that screening the transcription of tumour driver genes of ccRCC may possess diagnostical value in clinical oncology.

## Materials and Methods

### Patients and Specimens

The test cohort was composed of 60 freshly frozen samples derived from renal resections of 30 ccRCC patients provided by the Tumour Bank of the Department of Pathology, Albert Szent-Györgyi Medical School, University of Szeged. The sample size of the cohort was not determined statistically prior to experimentation. All diagnoses were histologically proven and classified according to the 2016 WHO classification of renal neoplasms [2]. Matched healthy tissue parts of the resected renal portions were also pathologically examined therefore considered as “normal” samples. Patient data are summarised in Table 1. All patients signed written informed consent forms. The study was performed with the approval of the Scientific and Research Ethical Committee of Hungarian Scientific Council (ETT TUKEB, #IV/5376-2/2020 EKU), and the experiments conform to the Declaration of Helsinki in 1995 (revised in Edinburgh in 2000).

**TABLE 1:** Summarisation of patient data of the cohort study.

Nr. of patient	Gender (Male/Female)	Age (year)	Tumour size (mm)	TNM classification	Distant metastases (No/Yes)
#1	M	63	33	pT1aNxM0	N
#2	F	55	32	pT1aMxM0	N
#3	M	55	30	pT1aNxM0	N
#4	M	68	40	pT1aNxM0	N
#5	M	78	39	pT1aNxM0	N
#6	F	68	25	pT1aNxM0	N
#7	F	61	30	pT1aNxM0	N
#8	F	59	45	pT1bNxM0	N
#9	M	78	56	pT1bNxM0	N
#10	M	73	44	pT1bNxM0	N
#11	M	50	52	pT1bNxM0	N
#12	F	40	51	pT1bNxM0	N
#13	M	39	72	pT2aNxM0	N
#14	M	78	60	pT3aNxM0	N
#15	F	40	42	pT3aNxM0	N
#16	M	77	34	pT3aNxM0	N
#17	F	71	125	pT3aNxMT	Y
#18	F	62	75	pT3aN1MT	Y
#19	M	66	62	pT3aNxM0	N
#20	F	51	35	pT3aNxM0	N
#21	F	60	125	pT3aNxM0	N
#22	F	66	95	pT3aNxM0	N
#23	F	68	72	pT3aNxMT	Y
#24	M	57	50	pT3aNxM0	N
#25	M	67	49	pT3aNxM0	N
#26	M	68	90	pT3aNxM0	N
#27	M	62	39	pT3aNxM0	N
#28	M	61	65	pT3aNxM0	N
#29	M	59	48	pT3aNxM0	N
#30	F	62	112	pT3aNxM0	N
Summarized	13 females	62.06 ± 10.68 years	57.57 ± 27.6 mm	12 pT1	3 with metastases
	17 males			1 pT2	27 wo metastases
				17 pT3	

### RNA and DNA Extraction, cDNA Synthesis

Total RNA isolates were prepared from fast-frozen tumour sections using Promega ReliaPrep Total RNA Miniprep System (Promega, Madison, WI), according to the manufacturer’s protocol. The amounts and purity of RNA preparations were analysed by NanoDrop Spectrophotometer (Thermo Fischer Scientific, Waltham, MA, United States). First-strand cDNA synthesis reactions were performed by SuperScript VILO cDNA Synthesis Kit (Invitrogen, Thermo Fischer Scientific, Waltham, MA, United States) using 400 ng of each RNA sample, according to the producer’s protocol.

Genomic DNA isolates were prepared from fast-frozen tumour sections using Macherey Nagel NucleoSpin Tissue Kit (Macherey Nagel, Düren, Germany), according to the manufacturer’s protocol. The amounts and purity of the DNA preparations were determined by NanoDrop Spectrophotometer (Thermo Fischer Scientific, Waltham, MA, United States), and 20 ng of each sample were used for further application.

### Quantitative Real-Time PCR

The qPCR reactions were performed on Thermo Scientific PIKO 96-well Thermal Cycler (Thermo Fischer Scientific, Waltham, MA, United States) platform using SYBR Green chemistry (GoTaq qPCR Master Mix, Promega, Madison, WI, United States). Sequences of gene-specific primer pairs are detailed in Table 2. Sample absolute gene expression and copy number variant (CNV) Cq values were normalised to the Cq average of *18S rRNA* and *cyclophilin B* internal control genes or an intergenic region, respectively. These values are referred to as normalised gene expression and normalised CNV in the text, respectively. Relative gene expressions and CNV values were calculated by the −ΔΔCq method referred to as relative gene expression and relative CNV in the text, respectively [16]. For the mRNA and CNV measurement of *VHL*, the same oligo pair was used in DNase treated RNA and RNA free gDNA samples, respectively.

**TABLE 2:** Sequences of PCR primers used in gene expression or CNV measurements.

Target	Forward primer (5′-3′)	Reverse primer (5′-3′)
*PBRM1*	TCA​GCC​CAT​TGA​CTT​GAT​GA	CCT​GAT​TGT​CTT​GCC​CAT​CT
*PBRM1* CNV	AGA​TCG​TAC​CGT​TGC​ACT​CC	GGA​ATA​CAG​TGG​CGG​GAT​CT
*BAP1*	CTC​GTG​GAA​GAT​TTC​GGT​GT	TCA​TCA​ATC​ACG​GAC​GTA​TCA
*BAP1* CNV	GCA​AAT​GTC​AGG​GGT​GAG​TG	CCT​GGC​ACT​GTC​TTC​CCT​AA
*SETD2*	GGA​AGA​ACA​GGG​ACG​ACA​GA	CTT​GAC​TTT​GGT​GGG​GAA​GA
*SETD2* CNV	TGC​CCA​GTG​TAG​TCT​CAA​TCC	GGC​AGC​AAA​GAG​GAC​GAT​AAG
*p14ARF*	GTGGCCCTCGTGCTGATG	GCGCTGCCCATCATCATG
*VHL*	TTG​TCC​GGA​GCC​TAG​TCA​AG	CAA​TGC​GCT​CCT​GTG​TCA​G
*18S rRNA*	CCT​GAG​AAA​CGG​CTA​CCA​CA	TTT​TCG​TCA​CTA​CCT​CCC​CG
*cyclophilin B*	CTT​CCC​CGA​TGA​GAA​CTT​CAA​ACT	CAC​CTC​CAT​GCC​CTC​TAG​AAC​TTT
intergenic region	TGG​AAC​TTC​TGG​AAG​ACA​CTG	TAC​ACC​ACT​CAA​GGG​AAA​CTG

### Statistical Analyses

Statistical comparisons of normal vs. tumour datasets were performed using SigmaPlot 12.5 software (Systat Software Inc., San Jose, CA, United States). Regression between the gene doses and gene expression was determined by using a Polynomial linear equation. The distribution of datasets was examined by Shapiro-Wilk normality and Equal Variance tests. The values of variances were determined by ANOVA or Kruskal-Wallis ANOVA on Ranks methods followed by multiple comparisons using Holm-Sidak test or Tukey-test, respectively. Diagnostic abilities and cutoff values were defined by the receiver operating characteristic curve (ROC curve) analysis tool of SigmaPlot 12.5 software using a 99% confidential interval.

## Results

### Copy Number Variations of *3p21* and *3p25* Loci Do Not Differentiate ccRCC and Normal Kidney Tissues

**FIGURE 1:**
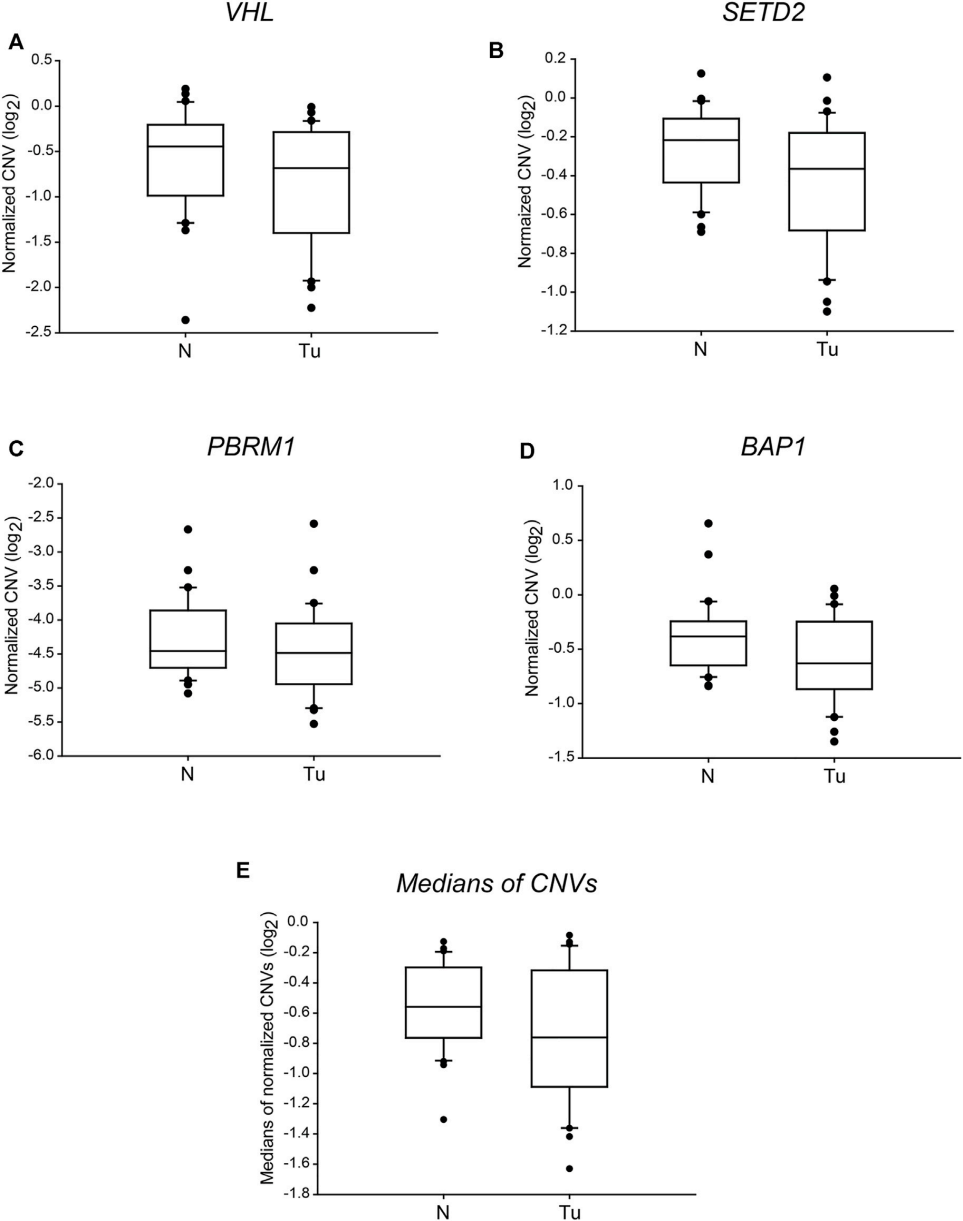
The copy numbers of the four tumour driver genes of *3p25* and *3p21* chromosomal loci are not significantly different in ccRCC tumour tissues than in normal kidney sections. Box plot graphs for comparison of normalised CNVs of each of the four somatic driver genes (panels **(A–D)**) or either the medians (panel **(E)**) of those in ccRCC tumour tissues and their normal counterparts (*n* = 60, 30 samples in each category). The Cq values of each gene were normalised to an intergenic region, indicated in log_2_ scale. Statistical analysis was performed using AVOVA. Legends: N, normal kidney tissues; Tu, ccRCC tumour samples.

Tumorigenesis of ccRCC is highly linked to alterations of somatic drivers of the *3p21* and *3p25* chromosome regions, namely *VHL*, *SETD2*, *PBRM1* and *BAP1* genes. These alterations include single nucleotide variations (SNVs), small insertions or deletions (INDELs), dinucleotide substitutions (DNVs) and somatic copy number alterations (SCNAs) like the deletion of chromosomal arm 3p as well as the alteration of DNA methylation patterns [8]. The loss of large genomic parts can be easily traced by determining the gene doses. Analysing the relative CNV results, we found double doses of *VHL* in two, and half gene doses in four ccRCC samples out of the 30 tumour samples tested (Table 4, column 3). Single copies of *SETD2* and *BAP1* were detected in three and six cases, respectively (Table 4 column 5 and 9, respectively). Finally, double doses of *PBRM1* were observed in four, and half in two samples. Most of these alterations afflicted only one gene out of the four; multiple gene hits were found exclusively in 2 cases (Table 4, column 7). However, we could not find any significant difference between the two categories when comparing the normalised CNVs or the medians of the four somatic drivers in ccRCC tumour tissues and their normal counterparts (Figures 1A–E). Our findings reflect that in most of the examined cases, the genetic alteration of chromosome 3p arm was other than large range deletion. Indeed, in some patients, point- and frameshift mutations, small deletions or insertions of *VHL* were proven by targeted sequencing (Table 3).

**TABLE 3:** Representative result of targeted VHL sequencing.

Nr. of patient	Detected mutation
#2	p.182_189del_RSLYEDLE
#8	V170Sfs*32
#11	S65fs*5
#14	146Lfs*13
#16	104_10 ins
#17	Y156T fs2
#19	D126S fs3
#22	p.163_168 del LQVVR
#25	c359 del G early stop

fs, frameshift; ins, insertion; del, deletion; early stop, early stop codon.

### Transcription Patterns Reflect Dysregulation of Somatic Driver Genes in ccRCC Tumours

Manifestation of genetic or epigenetic alterations of a given locus in transcript or protein levels can be regulated in multiple manners. In this sense, such dysregulation on genomic modifications could be the primary cause of impaired final cellular processes resulting incomplete cellular reprogramming. Thus, to better understand tumour behaviour, it would be necessary to identify genetic and even transcriptional changes occurring during cancer formation and progression. To carry out such a multilevel investigation, we measured the mRNA levels of *VHL*, *SETD2*, *PBRM1* and *BAP1* genes by qPCR on the same tumour-normal paired sample set used for our CNV characterisation. At first, we carried out a pairwise comparison of gene expressions in each patient sample. The relative mRNA levels of *VHL* showed a marked reduction in 24 out of the 30 tumours, which were not suspected by the data of gene copy numbers. In five patients among the six outliers, we did not detect a significant difference between (from 0.73- to 1.55-fold change) the tumorous and normal parts of the kidney. However, in one case, we measured a dramatic, 5.75-fold relative mRNA level increase of *VHL* in the ccRCC tissue sections despite that this patient had a normal copy number of *VHL* (Table 4, column 2 vs. 3). Similarly, the transcription of *PBRM1* was not altered, or just low level changed in 8 patients (between 0.73- and 1.59-fold change) and showed significant downregulation in 22 patients. As we found in the case of *VHL*, the relative mRNA levels of *PBRM1* did not correlate with the detected gene copy numbers (Table 4, column 6 vs. 7). A far more *BAP1* gene expression activity reduction was observed in 26 patient samples (Table 4, column 8 vs. 9). We also detected the parallel decrease of gene doses and transcription levels in only a few cases. The reduction of *SETD2* mRNA level was the most dramatic, with relative values between 0.04 and 0.49, which again did not reflect the detected copy numbers of the gene. Unaltered transcription was detected only in two patients (Table 4, column 4 vs. 5). Finally, we concluded that almost all the tested tumour samples showed reduced gene expression of at least one out of the four genes compared to their normal tissue pairs. Only one exceptional patient sample (patient #11) identified with unchanged and either upregulated transcription activity of *SETD2*, *PBRM1*, *BAP1* and *VHL*, respectively, in his ccRCC sections. Nevertheless, none of the general pathological tests showed any particular characteristic features that this ccRCC sample or the indicated patient possessed.

**TABLE 4:** Relative expression and -CNV values of *VHL*, *SETD2*, *PBRM1* and *BAP1* genes.

Nr. of patient	*VHL*		*SETD2*		*PBRM1*		*BAP1*	
	RE	CNV	RE	CNV	RE	CNV	RE	CNV
#1	0.25	0.57	0.15	0.75	0.55	0.92	0.33	0.61
#2	0.31	0.69	0.11	0.55	0.47	1.00	0.36	0.63
#3	0.37	1.13	0.29	0.99	0.50	0.79	0.47	0.93
#4	0.27	0.84	0.31	1.20	0.85	0.72	0.21	0.82
#5	0.21	1.01	0.08	1.08	0.18	0.63	0.12	1.29
#6	0.72	1.37	0.38	1.04	0.76	1.08	0.57	1.24
#7	0.13	0.81	0.13	0.71	0.29	0.83	0.23	0.98
#8	0.12	0.44	0.23	0.99	0.40	1.01	0.51	0.89
#9	0.57	0.73	0.26	0.90	0.41	0.74	0.46	0.77
#10	0.24	1.08	0.17	1.03	0.60	1.18	0.31	0.93
#11	5.75	1.11	1.32	0.73	1.59	2.11	0.75	0.80
#12	0.85	0.89	0.28	0.81	1.05	1.39	0.71	0.75
#13	0.37	0.85	0.23	1.24	0.95	1.49	0.48	0.66
#14	0.10	0.62	0.04	0.93	0.06	1.68	0.08	0.89
#15	0.40	0.83	0.20	0.92	0.60	0.44	0.35	0.73
#16	0.33	0.92	0.20	0.92	0.24	1.15	0.13	0.84
#17	0.43	0.60	0.10	0.57	0.24	1.27	0.12	0.57
#18	0.05	0.86	0.10	1.12	0.15	1.00	0.13	1.30
#19	0.33	1.64	0.11	0.64	0.24	0.47	0.20	1.23
#20	0.93	0.90	0.49	1.03	1.20	2.00	0.71	1.09
#21	1.55	1.59	1.22	0.93	1.38	1.41	0.83	0.86
#22	0.29	0.68	0.22	0.77	0.51	0.79	0.49	0.84
#23	0.46	0.48	0.33	0.55	0.28	0.97	0.37	0.65
#24	0.19	1.12	0.15	0.88	0.41	2.62	0.24	1.23
#25	0.73	0.66	0.22	0.77	0.73	0.74	0.41	0.68
#26	0.68	0.86	0.26	0.86	0.46	2.46	0.47	0.75
#27	0.31	0.94	0.12	0.78	0.55	1.35	0.19	0.77
#28	0.19	0.71	0.18	0.73	0.30	0.70	0.22	0.76
#29	0.47	0.75	0.23	1.15	0.51	0.70	0.38	0.94
#30	0.19	0.95	0.10	1.24	0.24	1.11	0.10	0.57

All the values are indicated as fold changes of mRNA levels detected in ccRCC samples compared to the normal kidney specimens of each patient.

RE, relative expression; CNV, copy number variant.

**FIGURE 2:**
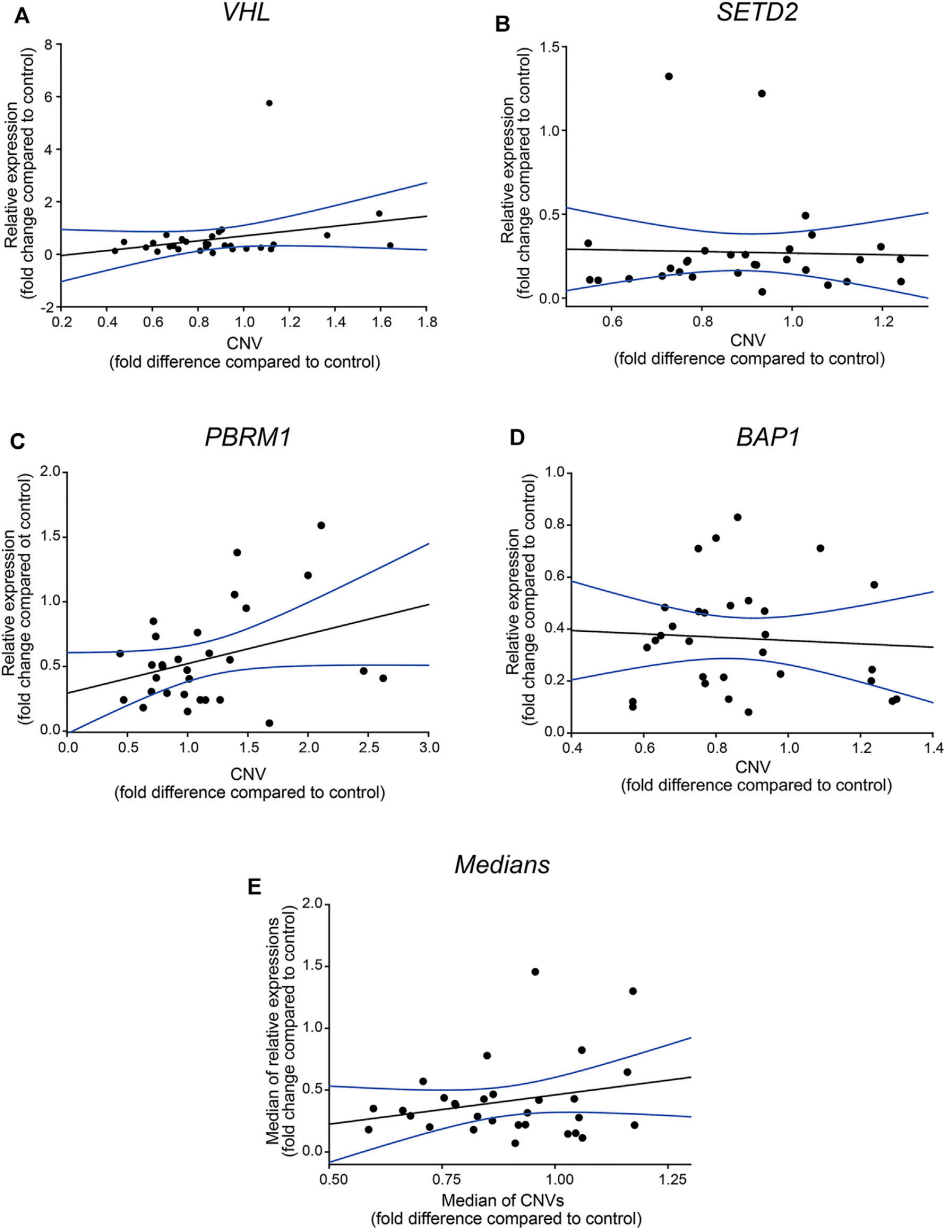
Relative gene expressions of *VHL*, *SETD2*, *PBRM1* and *BAP1* genes are not in correlation with CNVs values. Regression analysis of the correlation between the relative gene expressions of the four somatic driver genes (panel **(A–D)**) and their medians (panel **(E)**). The analyses were performed by SIGMA Plot program package, including ANOVA test. Relative gene expression and -CNV values were calculated using the −ΔΔCT method, indicated as fold-changes.

We performed regression analyses on each gene and the medians of the detected values to evaluate the statistical correlation between the detected relative gene expression and -CNV results. We found that none of the genes nor their medians showed a linear correlation between the relative transcription activity and gene doses (Figures 2A–E, respectively) which further verify that in most of the examined cases, the genetic alteration was other than large range deletion, such as epigenetic dysregulation or point mutation [17–19].

**FIGURE 3:**
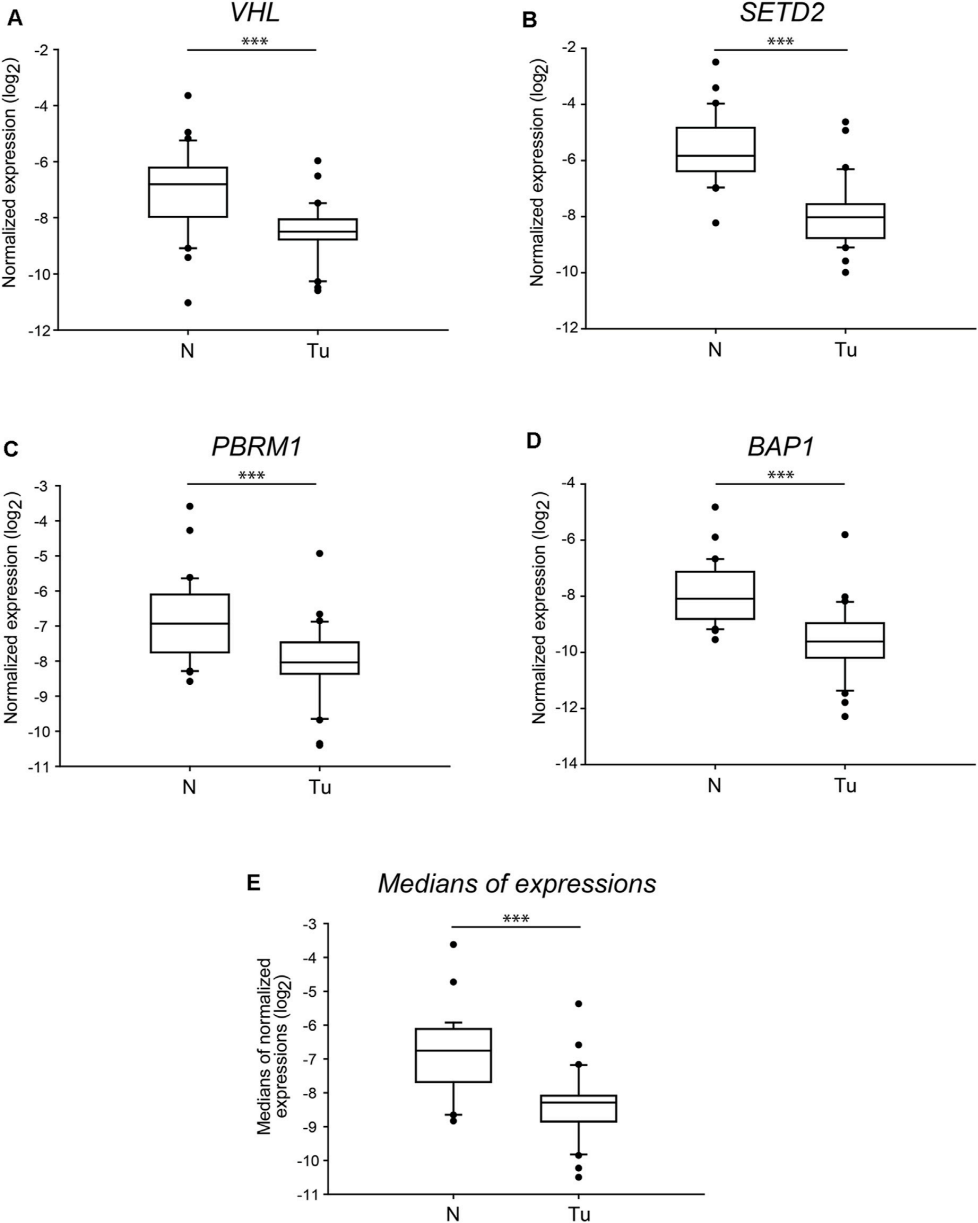
The normalised mRNA levels of the four tumour driver genes of *3p25* and *3p21* chromosomal loci are significantly different in ccRCC tumour tissues than in normal kidney sections. Box plot graphs for comparison of normalised gene expression of each of the four somatic driver genes (panels **(A–D)**) or either the medians (panel **(E)**) of those in ccRCC tumour tissues and their normal counterparts (*n* = 60, 30 samples in each category). The Cq values of each of the genes were normalised to the Cq average of housekeeping control genes, indicated in log_2_ scale. Statistical analysis was performed using AVOVA. Legends: N, normal kidney tissues; Tu, ccRCC tumour samples. ***p < 0.001.

To have a more specific sight of the manifested transcription alterations of the four genes, we also compared the normalised absolute gene expression levels detected in the normal and tumorous tissue samples. The distribution of the values indicated highly significant differences (*p* < 0.001) of each of the examined genes in control versus tumour comparisons (Figure 3). The mRNA levels of *PBRM1* showed a week overlap between the normal and tumorous samples (Figure 3C), while those of *BAP1* and *VHL* grouped more discretely (Figures 3D,A, respectively). Additionally, the transcription of *SETD2* was sharply discriminated between the two groups as the middle half of the gene expression values aggregated in distances, and only some extreme values overlayed (Figure 3B). The normalised mRNA level medians of the four genes were also significantly different in the tumorous samples compared to the normal ones with negligible overlapping (Figure 3E).

**FIGURE 4:**
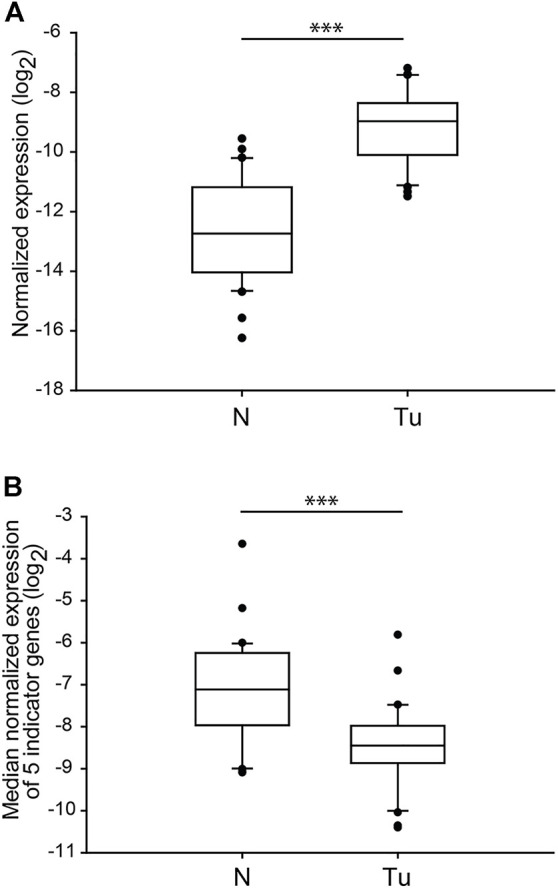
The normalised gene expression of *p14* ^*ARF*^ is significantly different in ccRCC tumour tissues than in normal kidney sections. Box plot graphs for comparison of normalised gene expression of *p14* ^*ARF*^ (panel **(A)**) or either the medians (panel **(B)**) of the five somatic driver genes in ccRCC tumour tissues and their normal counterparts (n = 60, 30 samples in each category). The Cq values of each p14ARF were normalised to the Cq average of housekeeping control genes, indicated in the log_2_ scale. Statistical analysis was performed using AVOVA. ***p < 0.001. Legends: N, normal kidney tissues; Tu, ccRCC tumour samples.

The loss or alteration of the *9p21.3* chromosome region, mainly affecting the gene of *cyclin-dependent kinase inhibitor 2A (CDKN2A)*, is highly associated with the formation of ccRCC originated metastatic lesions [9]. To test whether the gene expression of one of the several transcription variants of *CDKN2A*, *p14* ^*ARF*^ reflects ccRCC-associated changes, we determined and compared the normalised mRNA levels of each normal and tumour sample. Similarly to *SETD2*, we observed almost non-overlapping sharply different transcription levels between the two groups (*p* < 0.001) (Figure 4A). Nonetheless, dislike the genes of chromosome 3p, the relative gene expression of *p14* ^*ARF*^ showed dramatic overexpression in most of the tumorous samples (Table 5), indicating that almost all the patients were susceptible for metastases formation; however, the pathological records did not confirm this. The normalised gene expression medians of tumorous sections completed with the data of *p14* ^*ARF*^ were still significantly distinct from those of the normal tissues (*p* < 0.001) (Figure 4B). However, the values of the mRNA level medians of the four genes of 3p regions grouped noticeable more precisely in the two categories (Figure 3E vs. Figure 4B).

**TABLE 5:** Relative expression values of *p14* ^*ARF*^ gene.

Nr. of patient	Relative expression
#1	5.01
#2	6.06
#3	9.68
#4	10.70
#5	13.45
#6	19.09
#7	23.83
#8	1.21
#9	3.13
#10	6.75
#11	7.49
#12	152.22
#13	0.49
#14	2.46
#15	3.34
#16	5.21
#17	5.94
#18	7.24
#19	10.06
#20	16.56
#21	17.27
#22	20.68
#23	21.63
#24	28.34
#25	28.94
#26	29.45
#27	40.64
#28	49.01
#29	76.90
#30	102.89

All the values are indicated as fold changes of mRNA levels detected in ccRCC samples compared to the normal kidney specimens of each patient.

Our data indicate that the gene expression pattern derived from the transcription alteration of *3p21*, *3p25* and *9p21.3* chromosome regions is different in normal kidney and ccRCC tumour tissues. Particularly, examining mRNA levels of *VHL*, *SETD2*, *PBRM1,* and *BAP1* serve as discrete signatures for identifying ccRCC patients.

### Transcription Signatures of *3p21*, *3p25* and *9p21.3* Genomic Regions Are Valuable Indicators for ccRCC Diagnosis

**FIGURE 5:**
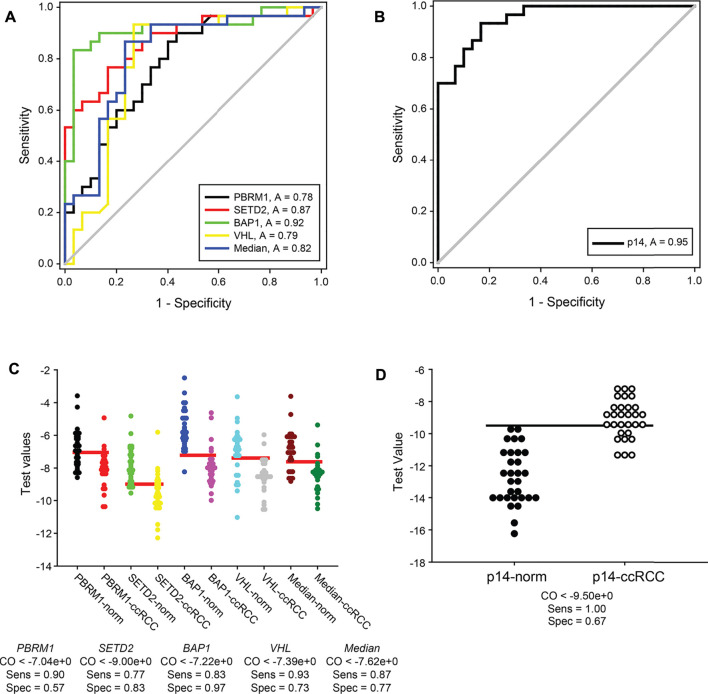
Normal kidney tissues and ccRCC tumour specimens can be sensitively distinguished by transcript signatures of *3p21*, *3p25* and *9p21.3* genomic regions. ROC curve analyses graphs of normalised gene expression each of *VHL*, *SETD2*, *PBRM1* and *BAP1* genes, their medians (panel **(A)**) and either of *p14* ^*ARF*^ (panel **(B)**). Calculated 1-specificity values are blotted in the function of sensitivity. ROC under area values are indicated in frames. The analyses were performed by SIGMA Plot program package. Dot blot graphs of Cutoff calculations of *VHL*, *SETD2*, *PBRM1* and *BAP1* gene expressions, their medians (panel **(C)**) and either of *p14* ^*ARF*^ (panel **(D)**). The data of normal kidney and ccRCC specimens are represented in pairs. Horizontal red and black lines (in panels **(C,D)**, respectively) show the calculated optimal cutoff values. Cutoff, sensitivity and specificity values are indicated for each of the genes and the medians under the graphs. Legends: norm, normal kidney tissues; ccRCC, ccRCC tumour samples; p14, *p14* ^*ARF*^; CO, cutoff; Sens, sensitivity; Spec, specificity.

Recently in tumour biology, many efforts have been made to identify valuable RNA signatures applied in diagnostics. Our results raised the further question of whether the transcription signature of *VHL*, *SETD2*, *PBRM1* and *BAP1* as a molecular marker has a valuable potential in ccRCC molecular diagnostics. We performed receiver operating characteristic (ROC) curves to calculate the sensitivity and specificity of the normalised gene expression data obtained from our cohort study (Figures 5A,B). Although *SETD2* was likely the most promising of the four genes, based on its data distribution (Figure 3B), the transcription pattern of *BAP1* showed the highest sensitivity and specificity, possessing a 0.92 area under the ROC curve. According to the statistical evaluation, measuring the mRNA level of *BAP1* is suitable for distinguishing ccRCC tumour specimens from normal kidney tissues with only an 8 per cent false rate (in 99% CI) (Figure 5A, green line). On the other hand, *SETD2* showed a modest utility in diagnosis with an 87% likelihood of categorising a given patient sample (Figure 5A, red line). Using *PBRM1* and *VHL* revealed the most inaccurate 22% and 21% false rates, respectively (Figure 5A, black and yellow lines, respectively). Regarding that, the distribution of the normalised gene expression data (box plots, see the previous section) is not in concert with the result of ROC analyses, using only the best candidate, *BAP1*, as a diagnostic marker may be oversimplified. As the four genes mapped on two distinct chromosomal regions of chromosome 3, applying the median normalised mRNA levels of *VHL*, *SETD2*, *PBRM1* and *BAP1* as transcription signatures is reasonable. Performing a ROC curve analysis on these datasets, 82% of sensitivity and specificity was revealed (Figure 5A, blue line).

As we proved that the gene expression of *CDKN2A* transcript variant, *p14* ^*ARF*^, could be the indicator of alteration of an independent genomic locus involved in the progression of tumorigenesis of ccRCC, we investigated the potential diagnostical value of it as well. The area under the ROC curve indicated that statistically, using the normalised mRNA level of *p14* ^*ARF,*^ 95% of the cases could be identified correctly (Figure 5B). Nevertheless, if the median values of gene expression were complemented with the data of *p14* ^*ARF*^ *,* the sensitivity and specificity of the test dropped down to 79% (data not shown). This surprising result could be that opposite to the significantly downregulated transcription of the four genes of the 3p chromosome, the mRNA levels of *p14* ^*ARF*^ were dramatically elevated in ccRCC tumours compared to the normal specimens mitigating the effectiveness of the test.

Using ROC curve analysis, possible diagnostical cutoff values can be evaluated as well. These cutoffs could serve as thresholds in the differentiation of normal kidneys from ccRCC tumorous tissues. The optimal cutoff values of *VHL*, *SETD2*, *PBRM1* and *BAP1* and their medians were determined from the pre-test probability and cost ratio by SigmaPlot to minimise negative and positive false results (Figure 5C). According to our results, *PBRM1* proved to be the less reliable marker. Only 57% of the samples could be sorted in the correct category, using the ideal threshold value of normalised absolute gene expression, −7.04 (Figure 5C first and second lanes). Surprisingly, the sensitivity and specificity of *SETD2* testing capacity (77% and 83%, respectively) statistically were predicted lower (Figure 5C second and third lanes) than expected based on the distribution of its expression. The highest sensitivity, 0.93, was evaluated for *VHL* with a cutoff value of −7.39. Nonetheless, determination of normalised mRNA level of *VHL* classifies only 73% of the specimens correctly (Figure 5C, seventh and eighth lanes). The testing ability of the transcription measurement of *BAP1* appeared to be the most reliable as it was also proved by its ROC curve. 83% of sensitivity can be achieved along with 97% of specificity, using the calculated cutoff, −9, of *BAP1* normalised mRNA levels as differentiation value which is the highest one among the four genes we tested (Figure 5C, fifth and sixth lanes). We believe that examining the transcription signature of the four genes encoded in *3p25* and *3p21* gene regions could serve as a more accurate indicator than determining the gene expression of a single but very reliable gene. For distinguishing ccRCC specimens from normal renal tissues, we calculated the cutoff value of median data of normalised mRNA levels. We found that using the cutoff as a threshold value (−7.62) of the median of the normalised transcription vales could discriminate the normal and cancerous categories with 87% of sensitivity and 77% of specificity, indicating a valuable possible diagnostic value of this approach (Figure 5C, ninth and tenth lane).

In our study, the transcription alteration of *p14* ^*ARF*^ was also a candidate for a diagnostic marker. Our findings showed that the mRNA level of *p14* ^*ARF*^ is significantly different in ccRCC specimens compared to normal kidney tissues. Thus, we determined the cutoff value of the *p14* ^*ARF*^ normalised gene expression dataset as well. We found that using the calculated cutoff (−9.5) as a testing threshold, we can reach 100% of sensitivity but only 65% of specificity statistically (Figure 5D). Furthermore, we showed that the transcription level of *p14* ^*ARF*^ was elevated in most of the ccRCC tumour samples opposite those of the four genes of *3p25* and *3p21* chromosome regions. For this reason, we suppose that the transcription evaluation of *p14* ^*ARF*^ might be used as an independent indicator as a supplementer for the gene expression signature of *VHL*, *SETD2*, *PBRM1* and *BAP1*.

Our results indicate that besides the single genetic alterations, epigenetic changes also have to be considered, which can occur during tumorigenesis, and are worth being characterised in multiple ways. Furthermore, observation of the transcriptional level of specific genes in the affected genomic loci can indicate the manifestation of mutations and chromosomal arrangements, as well as epigenetic dysregulation, which has a high impact on the entire behaviour of a given tumour. Therefore, we believe that monitoring the gene expression signature of tumour driver genes gives a unique opportunity to use more valuable diagnostic markers for supplementing the existing genetic testing.

## Discussion

The routine nucleic acid-based diagnosis mostly relies on the determination of DNA mutations, genomic rearrangements and alternative splice variants of different cancer types. However, these kinds of techniques are quite expensive and time-consuming. Therefore, finding new, cost-effective and rapid methods which serve reliable and predictive information is a clamant need of tumour pathology. In this study, we investigated the possible molecular diagnostic value of screening the transcription levels of tumour driver genes of ccRCC. Monitoring the copy numbers of four tumour suppressors of *3p25* and *3p21* chromosomal loci—*VHL*, *SETD2*, *PBRM1* and *BAP1*—we did not find a significant difference between the ccRCC tumorous and the normal adjacent kidney tissues indicating that the loss of the functions of these genes is frequently derived from other genetic alterations than deletion [7]. This hypothesis is indirectly proved by the findings of Bihr S. and colleagues, who recently showed that the protein expression of these genes and the global level of H3K36me^3^ are in correlation with the evolutionary subtypes of ccRCC [20]. We also found that the relative gene expressions of these genes did not correlate with the gene dosages. Additionally, our data also demonstrated that each of the absolute mRNA levels and the combined transcription profiles were significantly different in ccRCC samples than in normal kidney tissues. A similar phenomenon was observed in the case of the transcription variant of *CDKN2A*, *p14* ^*ARF*^. Performing ROC analysis, we proved that using the gene expression signature of driver genes encoded in *3p25* and *3p21* gene regions, ccRCC and normal kidney tissue samples can be discriminated with 87% sensitivity and 77% specificity. Since implicating the expression of *p14* ^*ARF*^ in this panel weakened the diagnostical value of that, we suppose that the transcription evaluation of *p14* ^*ARF*^ can be used as an independent indicator as a supplementer for the gene expression signature of *VHL*, *SETD2*, *PBRM1* and *BAP1*.

In recent years, many studies have been conducted to establish transcription-based methods for molecular tumour diagnosis. RNA as a biomarker possesses a great possibility for characterisation or even surveillance of a given tumour by providing dynamic information of tumour cells [21]. Regulatory RNAs, like microRNAs (miRNAs) or long non-coding RNAs (lncRNAs), are proven to regulate cell differentiation, proliferation, apoptosis, and consequently, if being dysregulated tumorigenesis, tumour progression or metastases can take place. The expression pattern of miRNAs has been shown to be useful in classifying tumours [22]. Multigene panels were established with proven reliability for cancer classification and prognoses, mainly using differential gene expression tools. A collection of 31 cell cycle regulation related genes was studied as progression markers in prostate cancer [23]. Defining the minimal set gene using microarray data, a 50 gene containing panel, PAM50, was identified and shown to be applicable in the classification of breast cancer and was getting used widely and now known as PROSIGNA [24]. Another similar test that is commercially available is the Oncotype DX Breast Recurrence Score Test. These gene panels were developed using comprehensive transcriptome analyses—regardless of genetic alterations—which are generally essential for identifying tumour-associated transcription features. Also, many efforts were taken to build mRNA- and miRNA-based assays for subtyping kidney carcinomas. These findings are similar in the sense that microarray data followed by hierarchical clustering analysis were used to determine gene- or miRNA sets distinguishing RCC and oncocytoma subtypes or stages [25–28]. Moreover, recently Wang Q and colleagues, reanalysing RNA microarray data, identified 11 co-expressed gene modules associated with the pathological stages and progression of ccRCC [29]. So did Wang Y et al. described fifteen hub-genes [30]. A comparable study by Chang L et al. identified a 3-mRNA-signature having a prognostic value [31]. Interestingly, none of *VHL*, *SETD2*, *PBRM1* and *BAP1* is indicated in these marker gene lists suspecting a missing link between the well-known DNA tumour markers and the actual behaviour of tumour cells.

## Conclusion

In summary, in this cohort study, we investigated the diagnostic value of the gene expression signature of known tumour driver genes of ccRCC. We showed that by calculating the median transcription values of the four ccRCC tumour suppressors of *3p25* and *3p21* chromosomal loci—*VHL*, *SETD2*, *PBRM1,* and *BAP1*–, complemented that of the *p14* ^*ARF*^, normal and ccRCC tissues can be distinguished. However, proving that these markers can be suitable in the staging of the disease has not been inspected yet. Our results also highlight the importance of examining the manifestation of the genetic alterations lying behind tumour progression.

The approach of this study was a first attempt to use genetic markers to predict transcription related alteration for diagnostics purposes. As ccRCC is one of the tumours having very well-defined oncogenic driver marker genes, we chose this cancer type to test our hypothesis. Our cohort study showed that, however, the specificity and sensitivity are quite moderate, the normal and ccRCC tissues can be distinguished based on the determination of transcriptional patterns using relatively simple qPCR measurements without using high-cost and time-consuming NGS approaches. As this technique requires much more attempt (including sample preparation, RNA extraction, specific instrumentation and training to perform quantitative PCR) than the general and widespread standard methods, like hematoxylin and eosin staining of sections followed by microscopic evaluation, we believe that this approach provides novel information for increasing the specificity of the diagnosis. In our study, the detected transcriptional variability of the adjacent non-tumorous tissues suspects that, despite the normal appearance of these tissue parts, there can be pre-neoplastic cells may contribute to the pathogenesis of ccRCC in a given patient [32].

## Data Availability

The original contributions presented in the study are included in the article/supplementary material, further inquiries can be directed to the corresponding author.
